# Diagnostic of Primary Ciliary Dyskinesia: guidelines to obtain appropriate ciliate cell samples

**DOI:** 10.1186/2046-2530-1-S1-P4

**Published:** 2012-11-16

**Authors:** S Blanchon, L Bassinet, A Clément, A Coste, E Escudier, C Thumerelle

**Affiliations:** 1 Dept Pediatric Pulmonology, Hôpital Trousseau, France; 2 Dept Pulmonary, Hôpital Inter-Communal, Creteil, France; 3 ENT Department, Hôpital Inter-Communal and Hôpital Henri Mondor, Creteil, France; 4 Dept Medical Genetic and Embryology, Hôpital Trousseau, France; 5 Dept Pediatrics, Hôpital Regional Universitaire, Lille, France

## Background

Primary Ciliary Dyskinesia (PCD) is a rare inherited disease (~1/20,000), characterized by ciliary structure/function abnormalities, responsible for impaired muco-ciliary transport, leading to recurrent upper and lower airways infections early in life and infertility. The diagnosis is confirmed on ciliate cell samples, collected by nasal and/or bronchial endoscopy. Patients usually need several samples, due to difficulties to get reliable results, especially during respiratory tract infections which are frequent in PCD. We created national guidelines aimed at obtaining the more efficient quality of ciliate cell samples for children and adults.

## Methods

A multicentric working group, created in 2009 within the French National Centre for Rare Respiratory Diseases, elaborated guidelines. It includes experimented ear-nose-throat and pulmonologist physicians, adults and children physicians, pathologists, technicians, involved in PCD diagnosis and care.

## Results

Improvement of sample quality requires 3 main duties: (i) 1 month free of respiratory tract infection or previous antibiotherapy (ii) preventing haemorrhagic complications (iii) a well-organized consignment to laboratory. We describe a step by step precise procedure for brushing (ciliary beat analysis) and biopsy (ultrastructural analysis) from nose and bronchus. The needed equipment for all procedures is provided, with references and seller’s contacts.

## Conclusion

Based on the French Reference Centre experience and on international recommendations, these new guidelines are very helpful to harmonize procedures and obtain efficient and usable ciliate cells sample. Application of these guidelines is an essential step to improve patient’s management, while reducing the number of samples and the delay to PCD diagnosis.

